# Effect of Auditory Input on Sensory Organization and Fall Risk in Young Adults With Hearing Aids

**DOI:** 10.1002/lary.32379

**Published:** 2025-07-04

**Authors:** Emre Orhan, Volkan Tutar, Bülent Gündüz, Recep Karamert, Mehmet Birol Uğur, Hakan Tutar

**Affiliations:** ^1^ Department of Audiology Gazi University Faculty of Health Sciences Ankara Turkey; ^2^ Department of Otorhinolaryngology Gazi University Faculty of Medicine Ankara Turkey

**Keywords:** auditory input, fall risk, postural control, sensory reweighting

## Abstract

**Objective:**

The aim of this study was to document postural control, sensory organization, and fall risk parameters of auditory input using computerized posturography in bilaterally hearing‐aided young adults.

**Materials and Methods:**

A total of 54 individuals aged 18 to 40 years participated in the study, including 36 bilateral hearing aid (HA) users and 18 normal‐hearing controls. HA users were divided into two groups based on the duration of device use: Group 1 included individuals using bilateral HAs for 12 months or less, and Group 2 included those using them for more than 12 months. The Sensory Organization Test (SOT) and computerized Fall Risk Assessment were administered to all participants meeting the inclusion criteria. Group 1 and Group 2 were assessed under both HA‐on and HA‐off conditions, while the control group was evaluated only under auditory stimulation.

**Results:**

SOT Condition 1, 2, and 4 scores of Group 1 in the HA‐on situation were statistically significantly higher than in the HA‐off situation. No statistically significant difference was observed in Condition 3, 5, and 6 scores. All SOT condition scores in the HA‐on situation of Group 2 were statistically significantly higher than those in the HA‐off situation. It was observed that the risk of falling performance was riskier when the HAs were turned off in Group 1 and Group 2 compared to when the HAs were turned on.

**Conclusion:**

It was observed that auditory input had a positive contribution to postural control and fall risk in young adult HA users.

**Level of Evidence:**

3.

## Graphical abstract

The aim of this study was to document postural control, sensory organization, and fall risk parameters of auditory input using computerized post urography in bilaterally hearing‐aided young adults. It was observed that auditory input had a positive contribution to postural control and fall risk in young adult hearing aid users.

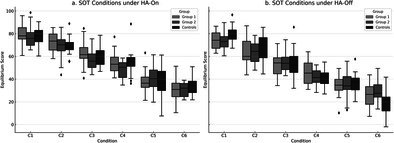

## 1 Introduction

In particular, over the last decade, the important role of auditory input in postural control has gained general support [1]. There is increasing evidence linking auditory input to postural control with hearing impairments. Notably, earlier studies have revealed that individuals with hearing impairments have poorer static postural control compared to those with normal hearing [2].

The relationship between auditory input deprivation and hearing loss on postural control has been explained in the literature by several theories. The close proximity of the auditory system and the vestibular system, the disruption of sensory inputs (visual, somatosensory, auditory, and vestibular) necessary for maintaining balance, and auditory input acting as a spatial anchor during balance maintenance have all been proposed as explanatory mechanisms [3, 4, 5, 6].

Previous studies examining the relationship between hearing and postural control have employed various methods ranging from quiet standing tasks to dynamic posturography systems [7]. More recent research has explored the contribution of auditory cues to postural stability using stimuli such as white noise or spatialized sound, and findings suggest that auditory input may support balance by enhancing spatial orientation or sensory integration [3, 8, 9, 10, 11]. Some studies have also examined the effect of hearing aid (HA) use on balance performance in individuals with hearing loss, highlighting the potential for auditory amplification to influence postural control [12]. Studies in the elderly have shown that auditory input deprivation is particularly reliant on somatosensory input. However, studies on young adults with hearing loss are limited [12, 13].

However, due to the inability to achieve complete homogenization in previous studies, the cause of hearing loss and the effect of auditory input on postural control have not yet been clearly understood. Failure to ensure homogeneity in age, degree of hearing loss, duration of HA use, and the lack of computerization in the posturographic equipment used to measure balance (e.g., the Sensory Organization Test [SOT]) has led to inconsistent results. The SOT, administered through computerized dynamic posturography (CDP), is a widely used tool for evaluating postural stability under systematically altered sensory conditions [14]. This test is particularly valuable for identifying the source and mechanism of balance dysfunction, as it allows clinicians and researchers to isolate failures in visual, vestibular, or somatosensory input processing, as well as the central adaptive mechanisms that integrate these cues [7, 15].

The SOT protocol is designed to quantitatively assess an individual's ability to maintain postural control using visual, proprioceptive, and vestibular information [14]. By systematically manipulating the reliability of visual and surface conditions, the test creates sensory‐conflicted environments. Under such conditions, individuals may exhibit poor postural strategies either due to ineffective use of sensory input or inappropriate adaptive responses [16]. For example, when both visual and proprioceptive feedback are disrupted, the test evaluates whether the person can still maintain their center of gravity using vestibular input alone [17]. The SOT's standardized format and rich output metrics make it a powerful method for exploring sensory reweighting and postural compensation strategies in both healthy and clinical populations [18].

The aim of this study was to document the effects of auditory input on parameters of postural control, sensory organization, and fall risk in bilateral hearing‐aided young adults who were homogeneous in age, gender, and degree of hearing loss, using computerized posturography.

## 2 Materials and Methods

### 2.1 Participants

Ethical approval for this study was approved by the Gazi University Non‐Interventional Clinical Research Ethics Committee (decision no: 2022/606). The current study included 36 individuals with bilateral HA and 18 controls aged between 18 and 40 years. The mean age of those with bilateral HA was 32.04 ± 3.2, and the mean age of controls was 31.75 ± 3.3. Participants with bilateral HA were divided into two groups according to the duration of HA use. Group 1 (*n* = 18, 9 male, 9 female), those who had used bilateral HA for 12 months or less. Group 2 (*n* = 18, 9 male, 9 female), those who had used bilateral HA for more than 12 months. Participants in the HA user groups (Groups 1 and 2) were all bilateral users with no prior history of unilateral amplification. The division into short‐term and long‐term users was based on existing literature suggesting that auditory and postural adaptation processes tend to stabilize after approximately 6–12 months of HA use [19]. Accordingly, a threshold of 12 months was used to differentiate participants with shorter versus longer exposure to consistent auditory input. In Group 1 (short‐term users), the average duration of HA use was 6 ± 1 months (median = 5 months), whereas in Group 2 (long‐term users), it was 27 ± 5 months (median = 23 months). All HA users reported full‐time use, defined as more than 8 h of daily use, and all were fitted with Receiver‐in‐Canal (RIC) or Receiver‐in‐the‐Ear (RITE) style devices.

Average hearing thresholds (mean of 0.25, 0.5, 1, 2, 3, 4, 6, and 8 kHz) for the controls were 6.75 dB HL (±1.25) for the right ear and 6.25 dB HL (±1.50) for the left ear. The hearing‐impaired group showed bilateral moderate degree (56–70 dB HL) sensorineural hearing loss: 61 dB HL (±5.45) for the right ear and 60.25 dB HL (±6.12) for the left ear. Peripheral vestibular end‐organ assessment included an evaluation of all six semicircular canals using the video head impulse test (vHIT) (ICS, GN Otometrics, Denmark), an evaluation of both saccules with the cervical vestibular evoked myogenic potential (cVEMP) (ICS, GN Otometrics, Denmark), and an evaluation of both utricles using ocular vestibular evoked myogenic potential (oVEMP) (ICS, GN Otometrics, Denmark). All participants had normal vestibular function (Table 1). Those with a history of orthopedic, neurological, cognitive, or visual impairment were excluded from the study.

**TABLE 1:** Comparison of vestibular tests of participants.

Test	Groups						
	Controls (*n* = 18)		Group 1 (*n* = 18)		Group 2 (*n* = 18)		
	Control (median)	Control (Q1–Q3)	Group 1 (median)	Group 1 (Q1–Q3)	Group 2 (median)	Group 2 (Q1–Q3)	*p*
cVEMP P13 Latency (Left, ms)	12.03	11.32–12.91	12.27	11.93–13.19	12.73	12.34–14.04	0.323
cVEMP N23 Latency (Left, ms)	23.11	21.75–24.05	23.87	21.37–25.09	22.13	20.79–23.88	0.528
cVEMP P13 Latency (Right, ms)	12.85	12.19–14.02	13.02	12.66–15.20	12.49	11.50–14.14	0.377
cVEMP N23 Latency (Right, ms)	21.34	20.04–23.06	21.66	20.09–24.32	21.49	19.93–23.67	0.637
cVEMP Amplitude (Left, μV)	49.54	38.49–69.93	45.29	28.75–64.14	47.58	34.19–60.47	0.448
cVEMP Amplitude (Right, μV)	52.76	40.89–64.30	59.97	45.27–69.10	50.28	36.74–62.24	0.379
oVEMP N10 Latency (ms)	12.19	11.61–13.64	12.53	12.13–12.74	11.84	11.01–12.56	0.151
oVEMP P25 Latency (ms)	25.99	25.46–26.43	25.81	25.48–26.35	26.2	25.77–26.59	0.133
oVEMP Amplitude (μV)	11.09	9.12–12.96	11.77	9.85–13.51	10.68	8.43–12.67	0.416
vHIT VOR Gain (Lateral Left)	0.91	0.86–0.94	0.92	0.88–0.96	0.94	0.89–0.97	0.317
vHIT VOR Gain (Lateral Right)	0.92	0.90–0.96	0.94	0.90–0.96	0.88	0.87–0.95	0.099
vHIT VOR Gain (LARP—Left Anterior)	0.85	0.79–0.92	0.82	0.76–0.90	0.86	0.81–0.93	0.666
vHIT VOR Gain (RALP—Right Anterior)	0.84	0.78–0.86	0.86	0.82–0.89	0.86	0.78–0.91	0.419
vHIT VOR Gain (LARP—Left Posterior)	0.84	0.79–0.89	0.85	0.79–0.90	0.83	0.79–0.89	0.453
vHIT VOR Gain (RALP—Right Posterior)	0.83	0.77–0.85	0.84	0.82–0.88	0.83	0.79–0.88	0.971

### 2.2 Methods

SOT and Fall Risk assessment were applied to participants via computerized posturography (Synapsys, France) who met the inclusion criteria.

#### 2.2.1 SOT

SOT was utilized to evaluate participants' ability to engage three sensory systems—somatosensory (SOM), vestibular (VEST), and visual (VIS)—that contribute to postural control [20]. The test consists of six progressively challenging conditions. In Condition 1, participants keep their eyes open (EO) while standing on a stable platform. Condition 2 involves eyes closed (EC) on the same stable platform. In Condition 3, with EO and a stable platform, a misleading visual stimulus (spider web) is introduced. Condition 4 requires participants to keep their EO on an unstable platform, while Condition 5 involves EC on an unstable platform. Finally, Condition 6 combines EO, an unstable platform, and a deceptive visual stimulus. The platform was designed to move in both the anteroposterior (AP) and mediolateral (ML) planes, with a separate assessment conducted for each plane.

The balance score was determined according to the participants' postural sway in the different test conditions. It was calculated as the following ratios: Condition 2 to Condition 1 for the somatosensory score, Condition 4 to Condition 1 for the visual score, and Condition 5 to Condition 1 for the vestibular score [21].

In addition to the sensory balance scores, preference (PREF) scores were also calculated. These scores assessed the participants' ability to ignore misleading visual information to maintain postural balance. Conditions 3 and 6 included visual input but were misleading and required participants to rely on somatosensory and vestibular information instead. The preference score was calculated by dividing the sum of Conditions 3 and 6 by the sum of Conditions 2 and 5. Finally, the global (GLOB) score was obtained by averaging all conditions [21].

#### 2.2.2 Fall Risk Assessment

In the fall risk assessment, participants' balance abilities were assessed during an external perturbation such as the SOT [22]. However, two different motion characteristics were used on the platform. In the translation plane, two different motion stimuli were used: instantaneous and sinusoidal. During the instantaneous movement, the energy expended by the participants to maintain their balance following the stimulus and the recovery times were obtained. The rate of postural responses (Gain) during the sinusoidal stimulus and the phase lag of postural sway in response to the sinusoidal stimulus were recorded [23]. “Energy” quantifies the overall sway effort of the individual to maintain balance during balance tasks. “Gain” reflects the amplitude ratio between the platform movement and the participant's sway. “Phase lag” represents the postural sway difference between the stimulus and the response, with larger values in degrees indicating delayed balance correction. “Recovery time” is defined as the time required to regain balance after a balance perturbation [22, 23].

#### 2.2.3 Procedure

Participants' balance tasks were performed in two conditions: with the hearing aids turned off (HA‐off) and with the hearing aids turned on (HA‐on), in the presence of white noise (0–20 kHz, 65 dB sound pressure level [SPL]) stimulus via a loudspeaker (frequency response of 0.8–20.0 kHz Model BT6900B Philips, Amsterdam, Holland) positioned at ear level 1 m behind the participants [11]. Ambient noise in the test room was measured using a sound level meter and was maintained at approximately 25–30 dB SPL. The choice of white noise was justified by its uniform spectral content and absence of semantic or spatial cues. Its application within the realm of postural control is based on earlier reports (e.g., Ross et al. [3]), which have evidenced its contribution in the modulation of balance performance by generalized auditory input. Rear placement of auditory stimuli has been used in previous studies examining sound‐induced modulation of postural control [3, 10, 24]. Gandemer et al. [8, 9] also emphasize the role of sound spatiality in balance behavior. By positioning the sound source behind the participant, it was aimed to deliver neutral, omnipresent auditory input without orienting effects, consistent with these established methodologies.

#### 2.2.4 Statistical Analysis

A priori power analysis was not conducted for this study. However, the sample size was informed by similar studies in the field examining postural control in hearing‐impaired populations under sensory manipulation conditions [5, 8, 11, 13]. Previous research with comparable group sizes (e.g., *n* = 15–20 per group) has demonstrated sufficient statistical sensitivity to detect group‐level differences in balance performance and sensory organization outcomes. The normality assumption of the data was examined both with the Shapiro–Wilk test and visually (histogram, scatter plot, kurtosis, skewness), and it was observed that the data did not meet the normality assumption. Analyses between three groups were conducted using the Kruskal–Wallis and Mann–Whitney U tests, and paired data within the group were analyzed with the Wilcoxon test. In order to control for Type I error across multiple comparisons, Bonferroni correction was applied to the resulting *p* values. For each set of related outcome variables (e.g., sensory scores, SOT conditions, fall risk parameters), the adjusted significance threshold was calculated by dividing the conventional alpha level (0.05) by the number of comparisons.

To assess the magnitude of observed effects, effect sizes (*r*) were calculated for non‐parametric comparisons, where applicable. For Wilcoxon tests, the effect size was computed using the formula *r = Z/√N*, and interpreted according to Cohen's thresholds: small (*r* = 0.1), moderate (*r* = 0.3), and large (*r* = 0.5). For Kruskal–Wallis or Mann–Whitney U comparisons, rank‐biserial correlation values were reported. Statistical significance was determined as *p* < 0.05. Analyses were performed with SPSS v.25 (IBM, USA).

## 3 Results

### 3.1 SOT

**FIGURE 1:**
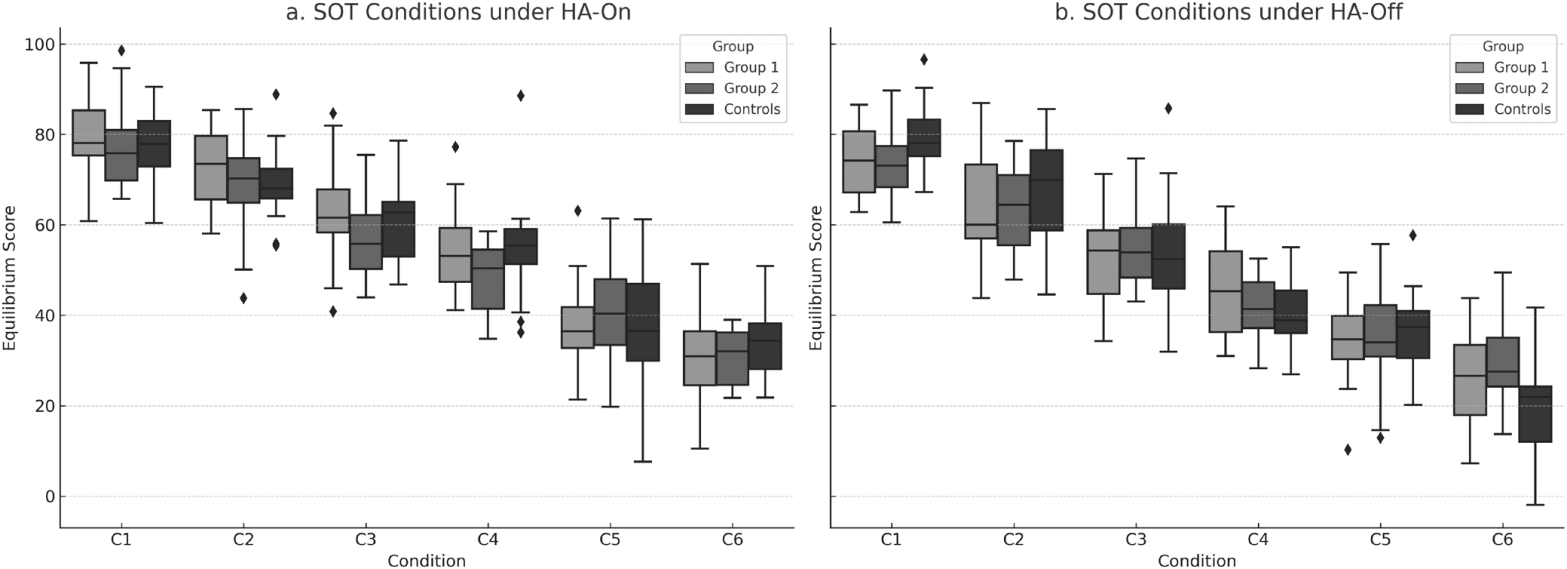
(a) Comparison of SOT condition scores of groups in the HA‐on condition. (b) Comparison of SOT condition scores of groups in the HA‐off condition. Boxplot comparisons of equilibrium scores across SOT conditions (C1–C6) under two settings: (a) with hearing aids (HA‐on), (b) without hearing aids (HA‐off). The boxplots show the median (center line), interquartile range (box limits), and 1.5 × IQR range (whiskers); outliers are represented by individual points. Statistical significance between groups was assessed using nonparametric tests.

Under the HA‐on condition, Kruskal–Wallis tests revealed significant group differences in SOT Conditions 1–4 (Figure 1). In Conditions 1–3, the control group had significantly higher equilibrium scores than both Group 1 and Group 2 (*p* < 0.001), with large effect sizes (Group 1: *r* = 0.680, 0.746, 0.649; Group 2: *r* = 0.694, 0.791, 0.757). In Condition 4, the control group scored higher than Group 1 (*p* = 0.006, *r* = 0.469), while the difference with Group 2 was not significant (*p* = 0.121, *r* = 0.259). No significant differences were observed in Conditions 5 and 6, with small or negligible effect sizes (e.g., *r* ≤ 0.179). Under the HA‐off condition (Figure 1b), significant differences were found in Conditions 1–3, 5, and 6. In Conditions 1–3, controls again scored significantly higher than both HA groups (*p* < 0.001), with large effect sizes (Group 1: *r* = 0.812, 0.854, 0.812; Group 2: *r* = 0.828, 0.854, 0.854). In Condition 5, the control group had higher scores than Group 1 (*p* = 0.013, *r* = 0.390) and Group 2 (*p* = 0.004, *r* = 0.480). In Condition 6, a significant difference was found only between Group 1 and Group 2 (*p* = 0.004, *r* = 0.480). No significant group differences were found in Condition 4 (*p* = 0.288), and effect sizes remained small (*r* = 0.22).

**FIGURE 2:**
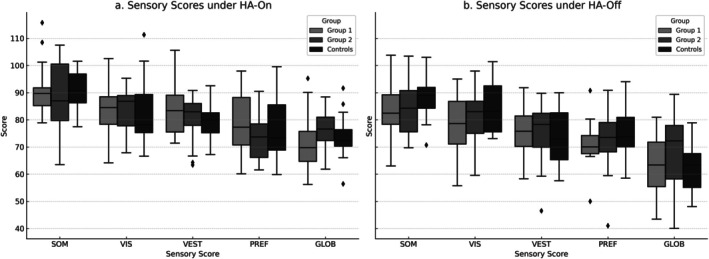
(a) Comparison of SOT sensory scores of groups in the HA‐on condition. (b) Comparison of SOT sensory scores of groups in the HA‐off condition. Boxplot comparisons of sensory analysis scores under SOT: (a) with hearing aids (HA‐on), (b) without hearing aids (HA‐off). The sensory ratios represent somatosensory (SOM), visual (VIS), vestibular (VEST), preference (PREF), and global (GLOB) scores. Each boxplot displays the median (center line), interquartile range (box), and 1.5 × IQR whiskers. Outliers are shown as individual points. Statistical significance across groups was tested with nonparametric methods.

Under the HA‐on condition, Kruskal–Wallis and post hoc Mann–Whitney U tests revealed significant group differences in several sensory scores (Figure 2a). The control group showed significantly higher SOM scores compared to Group 1 (*p* = 0.038, *r* = 0.346) and Group 2 (*p* = 0.011, *r* = 0.421), both with moderate effect sizes. VIS scores were significantly higher in the control group compared to both Group 1 (*p* < 0.001, *r* = 0.696) and Group 2 (*p* = 0.002, *r* = 0.512), with large effect sizes in both comparisons. For VEST and PREF scores, no statistically significant differences were observed between the groups (VEST: *p* = 0.613 and 0.217; PREF: *p* = 0.950 and 0.195), with negligible to small effect sizes (VEST: *r* = 0.084 and 0.206; PREF: *r* = 0.011 and 0.216). Regarding the GLOB scores, the control group scored significantly higher than Group 2 (*p* = 0.009, *r* = 0.438) and also showed a non‐significant trend compared to Group 1 (*p* = 0.164, *r* = 0.232). In the HA‐off condition, the control group had significantly higher SOM scores compared to both Group 1 (*p* = 0.016, *r* = 0.31) and Group 2 (*p* = 0.040, *r* = 0.27) (Figure 2b). Although the Kruskal–Wallis test indicated a statistically significant difference in VIS scores (*p* = 0.030), post hoc comparisons revealed no statistically significant group differences (*r* < 0.1). There were no significant differences between the groups for VEST (*p* = 0.589, *r* = 0.00) or PREF scores (*p* = 0.235, *r* = 0.10). However, the control group had significantly higher GLOB scores compared to both HA user groups (*p* < 0.001, *r* = 0.53), indicating a large effect size. In Group 1, significant improvements were observed in SOT Conditions 1, 2, and 4 with HAs on, while no changes were noted in Conditions 3, 5, and 6 (Table 2). In Group 2, all SOT condition scores improved significantly in the HA‐on condition (Table 2).

**TABLE 2:** SOT condition scores of participants with hearing aids.

Group 1 (*n* = 18)						
SOT conditions	HA‐on		HA‐off			
	Median	Q1–Q3	Median	Q1–Q3	Effect size (*r*)	*p*
Condition 1	85.8	81.95–89.25	76.55	70.88–77.69	0.12	**0.001**
Condition 2	83.25	79.70–87.50	72.18	69.08–75.31	*0.22*	**0.003**
Condition 3	73.43	63.95–78.35	57.44	53.73–67.66	0.25	0.085
Condition 4	79.6	76.20–82.05	65.16	59.32–72.39	0.41	**0.001**
Condition 5	51.1	43.45–63.20	43.66	38.83–49.92	0.36	0.064
Condition 6	35.3	20.65–47.55	35.93	28.16–42.42	0.28	0.777

Group 2 (*n* = 18)						
SOT conditions	HA‐on		HA‐off			
	Median	Q1–Q3	Median	Q1–Q3	Effect size (*r*)	*p*
Condition 1	85.25	80.20–88.40	76.92	72.84–79.00	0.18	**0.003**
Condition 2	81.38	71.50–83.60	72.46	69.57–75.34	0.27	**0.006**
Condition 3	68.68	59.60–71.90	61.95	55.94–64.54	0.32	**0.025**
Condition 4	74.33	72.00–79.00	67.06	57.35–71.84	0.39	**0.001**
Condition 5	54.3	41.00–62.15	43.3	36.92–48.13	0.48	**0.001**
Condition 6	32.25	21.20–42.10	26.72	22.22–34.00	0.44	**0.015**

*Note*: Bolded *p* values were less than 0.05.

Abbreviations: HA‐off, hearing aids turn off; HA‐on, hearing aids turn on; sd, standard deviation; SOT, Sensory organization test.

For sensory scores, Group 1 showed no significant differences between HA‐on and HA‐off in SOM, VIS, VEST, and PREF, although the GLOB score improved with HAs (Table 3). In Group 2, VEST and GLOB scores were higher in the HA‐on condition, while other sensory scores remained unchanged (Table 3).

**TABLE 3:** Sensory scores of Group 1 and Group 2.

Group 1 (*n* = 18)						
SOT sensory scores	HA‐on		HA‐off			
	Median	Q1–Q3	Median	Q1–Q3	Effect size (*r*)	*p*
SOM	97.72	93.82–100.00	93.01	89.60–100.00	0.29	0.100
VIS	93.42	86.58–100.00	87.66	75.74–96.01	0.18	0.076
VEST	57.94	51.07–70.67	58.93	45.55–69.50	0.31	0.901
PREF	79.10	66.24–87.31	82.88	92.31–67.45	0.14	0.470
GLOBAL	66.84	60.62–72.48	59.02	58.16–60.42	0.22	**0.007**

Group 2 (*n* = 18)						
SOT sensory scores	HA‐on		HA‐off			
	Median	Q1–Q3	Median	Q1–Q3	Effect size (*r*)	*p*
SOM	95.81	90.44–100.00	93.01	90.63–100.00	0.25	0.086
VIS	89.07	78.35–97.27	84.73	74.70–94.52	0.19	0.086
VEST	65.94	49.62–74.38	57.10	51.92–59.86	0.38	**0.022**
PREF	75.03	67.86–80.88	74.43	67.45–83.52	0.16	0.440
GLOBAL	64.67	61.98–69.24	57.40	54.88–59.22	0.35	**0.001**

*Note*: Bolded *p* values were less than 0.05.

Abbreviations: GLOB, global; HA‐off, hearing aids turn off; HA‐on, hearing aids turn on; PREF, preferential; sd, standard deviation; SOM, somatosensory; VEST, vestibular; VIS, visual.

### 3.2 Fall Risk Assessment

In the HA‐on condition, no significant differences were observed between groups under either eyes‐open or eyes‐closed conditions (Table 4). In the HA‐off condition, recovery times were shorter in controls than in both groups, both with EO and EC (Table 4).

**TABLE 4:** Fall risk comparison between groups.

Fall risk parameters of the HA‐on condition											
		Controls		Group 1		Group 2					
Test Condition	Parameter	Median	Q1–Q3	Median	Q1–Q3	Median	Q1–Q3	*p*	Effect size (*r*)	Post hoc significant comparisons	Adjusted *p*
Eyes open	Energy (mm^2^.s)	1911.50	1492.25–2440.75	1847.00	1492.25–2244.00	1680.50	1372.50–2238.75	0.856	< 0.1		
	Gain	0.69	0.57–0.85	0.67	0.57–0.89	0.75	0.62–0.91	0.521	< 0.1		
	Phase lag (°)	40.00	30.50–60.25	45.50	30.50–89.00	56.50	37.00–75.25	0.683	< 0.1		
	Recovery time (s)	5.10	3.71–6.37	5.52	3.85–6.37	5.04	4.52–5.99	0.928	< 0.1		
Eyes closed	Energy (mm^2^.s)	2128.50	1610.75–2729.25	2061.50	1610.75–2729.25	2142.00	1734.75–2553.75	0.987	< 0.1		
	Gain	1.08	0.93–1.38	1.17	0.96–1.39	1.08	0.97–1.29	0.810	< 0.1		
	Phase lag (°)	57.00	38.25–79.75	54.50	38.25–85.00	66.00	48.50–80.25	0.832	< 0.1		
	Recovery time (s)	4.86	3.88–6.00	4.86	3.88–6.14	4.70	3.81–5.68	0.645	< 0.1		

Fall risk parameters of the HA‐off condition											
Eyes open	Energy (mm^2^.s)	1911.50	1492.25–2440.75	1847.00	1492.25–2244.00	1680.50	1372.50–2238.75	0.054	0.275		
	Recovery time (s)	5.10	3.71–6.37	5.52	3.85–6.37	6.66	5.85–10.6	**0.014**	0.354	Contols‐Group 1 Controls‐Group 2	**0.031** **0.038**
	Gain	0.69	0.57–0.85	0.67	0.57–0.89	0.75	0.62–0.91	0.050	0.287		
	Phase lag (°)	40.00	30.50–60.25	45.50	30.50–89.00	56.50	37.00–75.25	0.038	0.298		
Eyes closed	Energy (mm^2^.s)	2128.50	1610.75–2729.25	2466.99	2060.96–2889.57	2609.54	2032.34–3150.63	0.135	0.198		
	Recovery time (s)	4.86	3.88–6.00	6.98	6.60–7.19	6.32	5.12–7.11	**< 0.001**	0.531	Contols‐Group 1 Controls‐Group 2	**< 0.001** **0.032**
	Gain	1.08	0.93–1.38	1.12	0.92–1.31	1.18	0.84–1.51	0.941	< 0.1		
	Phase lag (°)	57.00	38.25–79.75	73.47	70.16–84.31	76.21	63.16–83.18	0.071	0.254		

*Note*: Bolded *p* values were less than 0.05.

Abbreviations: HA‐off, hearing aids turn off; HA‐on, hearing aids turn on.

Within‐group comparisons showed that in Group 1, recovery time was significantly longer in the HA‐off condition, particularly under eyes‐closed settings, while other fall risk parameters remained similar (Table 5). In Group 2, energy expenditure and recovery time increased in the HA‐off condition with EO, and recovery time also increased under eyes‐closed conditions (Table 5).

**TABLE 5:** Fall risk findings of Group 1 and Group 2.

Group 1							
		HA‐on		HA‐off			
	Fall risk	Median	Q1–Q3	Median	Q1–Q3	Effect size (*r*)	*p*
Eyes open	Energy (mm^2^.s)	1847	1420–2287	2285.74	1921.95–2669.85	0.20	0.396
	Recovery time (s)	5.52	3.60–6.49	8.53	4.59–10.04	0.49	**0.035**
	Gain	0.67	0.57–0.89	1.21	0.39–2.67	0.35	0.133
	Phase lag (°)	45.5	30–98	81.64	47.40–103.02	0.45	0.053
Eyes closed	Energy (mm^2^.s)	2061	1590–2769	2466.9	2036.58–2903.36	0.34	0.145
	Recovery time (s)	4.86	3.67–6.17	6.98	6.53–7.21	0.69	**0.003**
	Gain	1.17	0.96–1.39	1.12	0.92–1.31	0.08	0.710
	Phase lag (°)	54.5	36–86	73.47	69.88–84.38	0.64	**0.006**

Group 2							
		HA‐on		HA‐off			
	Fall Risk	Median	Q1–Q3	Median	Q1–Q3	Effect size (*r*)	*p*
Eyes open	Energy (mm^2^.s)	1680	1319–2254	2672.25	2240.18–2905.53	0.75	**0.001**
	Recovery time (s)	5.04	4.45–6.02	6.66	5.85–10.6	0.56	**0.016**
	Gain	0.75	0.62–0.91	0.09	−1.3‐1.65	0.42	0.071
	Phase lag (°)	56.5	32–79	71.94	54.19–122.65	0.40	0.085
Eyes closed	Energy (mm^2^.s)	2142	1716–2558	2609.54	1986.63–3182.79	0.43	0.064
	Recovery time (s)	4.7	3.77–5.68	6.32	5.07–7.14	0.58	**0.014**
	Gain	1.08	0.96–1.32	1.18	0.82–1.51	0.04	0.840
	Phase lag (°)	66	48–82	76.21	62.05–84.61	0.35	0.133

*Note*: Bolded *p* values were less than 0.05.

Abbreviations: HA‐off, hearing aids turn off; HA‐on, hearing aids turn on.

## 4 Discussion

The aim of this study was to determine the risk of sensory organization skills decline by examining the postural control skills of young adult HA users under different environmental perturbations.

Previous studies have suggested that auditory input may either improve or impair postural control [8, 10, 24, 25]. However, the relationship between auditory information, hearing loss, and balance remains unclear, largely due to methodological heterogeneity [2]. Many studies included participants with varying ages, degrees of hearing loss, and unexcluded vestibular pathologies, which may have confounded the results. In contrast, the present study ensured homogeneity by selecting participants with similar hearing loss profiles and age range, and by objectively excluding vestibular dysfunction. This allowed for a clearer assessment of auditory input effects in individuals with isolated hearing loss.

Although the SOT is not specifically designed to assess auditory input, it remains a validated tool for evaluating postural control under controlled sensory conflict. In this study, auditory input via HAs was used not to isolate an auditory pathway, but to examine its potential role in modulating central sensory weighting. In conditions where somatosensory or visual input is compromised (e.g., Conditions 4 and 5), the test allows exploration of whether auditory cues act as compensatory inputs or influence multisensory integration. Changes in postural performance may thus reflect both balance improvements and adaptations in sensory processing.

Kanegaonkar et al. [5] demonstrated that reducing auditory input in individuals with normal hearing, using ear protection, increased postural sway, underscoring the role of sound in balance control. Similarly, Maheu et al. [12] reported that HAs improved postural sway in individuals with both hearing and vestibular impairments, suggesting a compensatory role for auditory input when other sensory systems are compromised [12, 26].

Group 1 had higher SOT scores in Conditions 1, 2, and 4 with HAs on compared to off. Moderate effect sizes were observed in Conditions 4 and 5 under the HA‐on condition. These results suggest that auditory input may begin to support sensory reweighting and postural control even in the early stages of HA use. However, changes in vestibular and global sensory scores were limited, possibly due to insufficient time for full central adaptation.

Group 2, consisting of experienced HA users, showed higher SOT scores with HAs on. The strongest effects appeared in Conditions 5 and 6, where moderate to large effect sizes indicated improved balance under sensory conflict, particularly when visual and somatosensory inputs were unreliable. These results suggest that long‐term auditory input may enhance central sensory reweighting and support more efficient postural strategies. Previous studies have indicated that healthy young adults are more likely to rely on auditory cues for balance in situations with reduced sensory input, such as standing with EC or on a firm surface [2]. However, this was not observed when standing on foam among healthy young adults [9]. In contrast, foam played a role in integrating auditory cues for individuals with vestibular and hearing loss [12]. There may be several reasons for the differences between the results of previous studies and the present study. First, the majority of the young adult populations in the previous studies were normal hearing. Another possible reason may be differences in the posturographic measurements and standardized tests used.

When the sensory organization was examined, there was no change in the sensory scores of Group 1, but the vestibular score of Group 2 decreased when the HA was turned off. Previous studies have noted sensory reweighting, particularly in somatosensory perception. However, differences in age group, degree of hearing loss, and standardized test batteries used to assess postural control in previous studies should be considered. There was a previous study using foam pads in young adult studies that showed no sensory bias. This suggests that foam may not be challenging enough to trigger sensory reweighting in healthy young adults compared to those with sensory impairments [27]. The difference in postural performance between Group 1 and Group 2 under the HA‐on condition can be attributed to the duration of HA use. Although both groups were tested with auditory input, Group 2 participants had over 12 months of HA experience, likely supporting greater neural adaptation and more efficient integration of auditory cues into postural control. In contrast, Group 1 participants, with ≤ 12 months of device use, may not yet have developed the same level of multisensory adaptation. These findings suggest that the benefits of auditory input on balance may depend not only on the presence of HAs but also on the cumulative effect of auditory exposure over time.

Despite the reduction of somatosensory and visual input in certain SOT conditions, no increased reliance on auditory cues was observed. This suggests that white noise, a non‐directional and non‐spatial auditory stimulus, may not be salient enough to be prioritized in the central sensory reweighting process [28, 29, 30]. Instead, it may serve as a general stabilizing reference without being actively weighted when other sensory channels are compromised. These findings indicate that auditory input plays a background integrative role in postural control rather than compensating for the loss of visual, vestibular, or proprioceptive input. While previous studies have demonstrated that auditory cues contribute to balance, their influence appears more limited compared to dominant sensory modalities such as vision, proprioception, and vestibular input [3, 5, 7, 9, 31].

It was difficult to make a quantitative fall risk assessment based on postural control assessments in previous studies. A standardized fall risk assessment was performed in the current study. Group 1 was observed to have delayed responses to predictable and unpredictable perturbations to maintain their balance when the HAs were off. Notably, recovery time under EC conditions showed a large effect size, indicating that participants were able to regain postural stability more effectively when auditory cues were available. However, as no meaningful change was observed in energy expenditure, it is possible that postural corrections were improved in timing, without an increase in physical effort. These findings suggest that even early exposure to auditory cues can enhance balance timing mechanisms, though more substantial multisensory integration may require prolonged HA experience. Group 2 was observed to have the same delayed responses and to expend more energy to maintain their balance when the HAs were off. With HAs on, there were no differences in fall risk assessment parameters between HA users and controls, indicating that individuals were not at risk of falling when HAs were on. It was difficult to discuss the data because previous studies assessed fall risk mostly in older adults [1, 32]. In terms of fall risk, recovery time showed a medium effect size and while energy values increased slightly, the clinical interpretation of this change is nuanced. An increase in energy expenditure may indicate a fall risk but may also reflect a more active postural control strategy, potentially driven by increased awareness or reactivity rather than instability. However, the findings in the current study with HAs off suggested that age‐related hearing loss and auditory input deprivation may contribute to an increased risk of falling.

### 4.1 Limitations and Future Directions

Although this study employed nonparametric frequentist analyses due to the characteristics of the data and sample size, future studies with larger and more complex datasets may benefit from the use of multilevel or Bayesian statistical models. These approaches could provide a more detailed understanding of individual‐level variability and condition‐specific effects in the context of sensory integration and postural control.

## 5 Conclusion

In the current study, the effects of hearing loss and auditory input on postural control skills, sensory organization, and fall risk in young adult HA users were examined using standardized posturographic test methods. Balance skills in young adult hearing loss participants were documented by attempting to homogenize in terms of age, gender, and hearing loss range. It was observed that auditory input had a positive contribution to postural control and fall risk in young adult HA users. Future studies can provide homogenization in terms of etiology in different age groups and reveal the effects of auditory input and hearing loss on postural control more clearly.

## Conflicts of Interest

The authors declare no conflicts of interest.

## Data Availability

Data can be provided via zenodo (10.5281/zenodo.15408166) upon request.

## References

[1] L. Behtani , D. Paromov , K. Moïn‐Darbari , et al., “Sensory Reweighting for Postural Control in Older Adults With Age‐Related Hearing Loss,” Brain Sciences 13 (2023): 1623. 38137071 10.3390/brainsci13121623 PMC10741952

[2] A. V. Lubetzky , M. Gospodarek , L. Arie , J. Kelly , A. Roginska , and M. Cosetti , “Auditory Input and Postural Control in Adults: A Narrative Review,” JAMA Otolaryngology. Head & Neck Surgery 146 (2020): 480–487. 32163114 10.1001/jamaoto.2020.0032

[3] J. M. Ross , O. J. Will , Z. McGann , and R. Balasubramaniam , “Auditory White Noise Reduces Age‐Related Fluctuations in Balance,” Neuroscience Letters 630 (2016): 216–221. 27495013 10.1016/j.neulet.2016.07.060

[4] J. Vitkovic , C. Le , S. L. Lee , and R. A. Clark , “The Contribution of Hearing and Hearing Loss to Balance Control,” Audiology and Neurootology 21 (2016): 195–202. 10.1159/000445100 27251708

[5] R. G. Kanegaonkar , K. Amin , and M. Clarke , “The Contribution of Hearing to Normal Balance,” Journal of Laryngology and Otology 126 (2012): 984–988. 22906584 10.1017/S002221511200179X

[6] M. Maheu , S. Pagé , A. Sharp , A. Delcenserie , and F. Champoux , “The Impact of Vestibular Status Prior to Cochlear Implantation on Postural Control: A Multiple Case Study,” Cochlear Implants International 18 (2017): 250–255. 28665247 10.1080/14670100.2017.1341362

[7] R. J. Peterka , “Sensorimotor Integration in Human Postural Control,” Journal of Neurophysiology 88 (2002): 1097–1118. 12205132 10.1152/jn.2002.88.3.1097

[8] L. Gandemer , G. Parseihian , R. Kronland‐Martinet , and C. Bourdin , “The Influence of Horizontally Rotating Sound on Standing Balance,” Experimental Brain Research 232 (2014): 3813–3820. 25146572 10.1007/s00221-014-4066-y

[9] L. Gandemer , G. Parseihian , R. Kronland‐Martinet , and C. Bourdin , “Spatial Cues Provided by Sound Improve Postural Stabilization: Evidence of a Spatial Auditory Map?,” Frontiers in Neuroscience 11 (2017): 357. 28694770 10.3389/fnins.2017.00357 PMC5483472

[10] R. D. Easton , A. J. Greene , P. DiZio , and J. R. Lackner , “Auditory Cues for Orientation and Postural Control in Sighted and Congenitally Blind People,” Experimental Brain Research 118 (1998): 541–550. 9504849 10.1007/s002210050310

[11] V. Tutar , İ. T. Batuk , and M. Özbal Batuk , “The Impact of Auditory Input on Postural Control in Adults With Unilateral Cochlear Implants,” Audiology and Neurotology 30 (2025): 263–271. 39799935 10.1159/000543402

[12] M. Maheu , L. Behtani , M. Nooristani , et al., “Vestibular Function Modulates the Benefit of Hearing Aids in People With Hearing Loss During Static Postural Control,” Ear and Hearing 40 (2019): 1418–1424. 30998550 10.1097/AUD.0000000000000720

[13] M. Maheu , A. Sharp , S. P. Landry , and F. Champoux , “Sensory Reweighting After Loss of Auditory Cues in Healthy Adults,” Gait & Posture 53 (2017): 151–154. 28157577 10.1016/j.gaitpost.2017.01.015

[14] J. M. Furman , “Posturography: Uses and Limitations,” Baillière's Clinical Neurology 3 (1994): 501–513. 7874405

[15] L. M. Nashner and G. McCollum , “The Organization of Human Postural Movements: A Formal Basis and Experimental Synthesis,” Behavioral and Brain Sciences 8 (1985): 135–150.

[16] J. M. Furman , “Role of Posturography in the Management of Vestibular Patients,” Otolaryngology and Head and Neck Surgery 112 (1995): 8–15. 10.1016/S0194-59989570300-4 7816461

[17] E. Orhan , İ. T. Batuk , and M. O. Batuk , “Balance Performance in Young Adults With Hearing Aids: How Can It Be Affected by the Visual Cognitive Task?,” Journal of Speech, Language, and Hearing Research 67 (2024): 2774–2781. 10.1044/2024_JSLHR-23-00530 39018264

[18] E. Orhan , B. Altın , and S. Aksoy , “Effect of Smartphone Use on Static and Dynamic Postural Balance in Healthy Young Adults,” American Journal of Audiology 30 (2021): 703–708. 34297603 10.1044/2021_AJA-20-00210

[19] H. Glick and A. Sharma , “Cross‐Modal Plasticity in Developmental and Age‐Related Hearing Loss: Clinical Implications,” Hearing Research 343 (2017): 191–201. 27613397 10.1016/j.heares.2016.08.012 PMC6590524

[20] M. B. Badke , J. A. Miedaner , C. R. Grove , T. A. Shea , and G. M. Pyle , “Effects of Vestibular and Balance Rehabilitation on Sensory Organization and Dizziness Handicap,” Annals of Otology, Rhinology and Laryngology 114 (2005): 48–54. 15697162 10.1177/000348940511400109

[21] E. T. Şahin , E. Orhan , V. Tutar , H. Tutar , and B. Gündüz , “The Effect of Different Sensory Perturbations on Postural Control and Fall Risk in Benign Paroxysmal Positional Vertigo Patients,” American Journal of Audiology 33 (2024): 874–881. 39052352 10.1044/2024_AJA-23-00263

[22] V. Ghulyan‐Bedikian and M. Paolino , “Comparative Study of Dynamic Balance in Fallers and Non‐Fallers,” French Otorhinolaryngology 88 (2005): 89–96.

[23] V. Ghulyan‐Bedikian and M. Paolino , “Posturography for Evaluating Risk of Falls in Elderly Unstable Patients,” 2005.

[24] S. A. Raper and R. W. Soames , “The Influence of Stationary Auditory Fields on Postural Sway Behaviour in Man,” European Journal of Applied Physiology and Occupational Physiology 63 (1991): 363–367. 1773813 10.1007/BF00364463

[25] S. H. Park , K. Lee , T. Lockhart , and S. Kim , “Effects of Sound on Postural Stability During Quiet Standing,” Journal of Neuroengineering and Rehabilitation 8 (2011): 67. 22168248 10.1186/1743-0003-8-67 PMC3275494

[26] K. Rumalla , A. M. Karim , and T. E. Hullar , “The Effect of Hearing Aids on Postural Stability,” Laryngoscope 125 (2015): 720–723. 25346316 10.1002/lary.24974

[27] A. Lubetzky‐Vilnai , S. W. McCoy , R. Price , and M. A. Ciol , “Young Adults Largely Depend on Vision for Postural Control When Standing on a BOSU Ball but Not on Foam,” Journal of Strength and Conditioning Research 29 (2015): 2907–2918. 26402476 10.1519/JSC.0000000000000935

[28] K. Anton , A. Ernst , and D. Basta , “A Static Sound Source Can Improve Postural Stability During Walking,” Journal of Vestibular Research 31 (2021): 143–149. 33492257 10.3233/VES-200015

[29] C. Guigou , M. Toupet , B. Delemps , S. Heuschen , S. Aho , and A. Bozorg Grayeli , “Effect of Rotating Auditory Scene on Postural Control in Normal Subjects, Patients With Bilateral Vestibulopathy, Unilateral, or Bilateral Cochlear Implants,” Frontiers in Neurology 9 (2018): 972. 30505289 10.3389/fneur.2018.00972 PMC6250812

[30] X. Zhong and W. A. Yost , “Relationship Between Postural Stability and Spatial Hearing,” Journal of the American Academy of Audiology 24 (2013): 782–788. 24224986 10.3766/jaaa.24.9.3

[31] P. F. Smith , “Hearing Loss Versus Vestibular Loss as Contributors to Cognitive Dysfunction,” Journal of Neurology 269 (2022): 87–99. 33387012 10.1007/s00415-020-10343-2

[32] D. Basta , L. Borsellino , K. Anton , and A. Ernst , “Influence of Auditory Information on Postural Control During Different Gait Tasks in the Elderly,” Journal of International Advanced Otology 19 (2023): 22–27. 36718032 10.5152/iao.2023.22671 PMC9984904

